# The N^6^-methyladenosine-mediated lncRNA WEE2-AS1 promotes glioblastoma progression by stabilizing RPN2

**DOI:** 10.7150/thno.74600

**Published:** 2022-08-29

**Authors:** Boyan Li, Rongrong Zhao, Wei Qiu, Ziwen Pan, Shulin Zhao, Yanhua Qi, Jiawei Qiu, Shouji Zhang, Qindong Guo, Yang Fan, Hao Xu, Ming Li, Gang Li, Hao Xue

**Affiliations:** 1 Department of Neurosurgery, Qilu Hospital, Cheeloo College of Medicine and Institute of Brain and Brain-Inspired Science, Shandong University, Jinan 250012, Shandong, China.; 2 Shandong Key Laboratory of Brain Function Remodeling, Jinan 250012, Shandong, China.

**Keywords:** Glioblastoma, N6-methyladenosine, IGF2BP3, RPN2, ubiquitination

## Abstract

**Background:** Glioblastoma (GBM) is the most common primary brain malignancy and has high aggressiveness and a poor prognosis. N6-methyladenosine (m6A) represents the most prevalent methylation modification of lncRNAs and has been shown to play important roles in the pathophysiological processes of tumors. However, the distribution and function of m6A modifications in lncRNAs in GBM tissues have not been fully revealed.

**Methods:** The global depiction of m6A-modified lncRNA expression patterns in GBM tumor tissues was screened via m6A high-throughput sequencing. Gain- and loss-of-function assays were performed to investigate the role of WEE2-AS1 in GBM. Mass spectrometry and RNA-pulldown, RNA immunoprecipitation (RIP), luciferase reporter and coimmunoprecipitation assays were performed to explore the mechanism of m6A-mediated upregulation of WEE2-AS1 expression and the downstream mechanism promoting the malignant progression of GBM.

**Results:** Herein, we report the differential expression profile of m6A-modified lncRNAs in human GBM tissues for the first time. WEE2-AS1 was identified as a novel m6A-modified lncRNA that promotes GBM progression and was post-transcriptionally stabilized by IGF2BP3, an m6A reader. Moreover, we confirmed that WEE2-AS1 promoted RPN2 protein stabilization by preventing CUL2-mediated RPN2 K322 ubiquitination, thereby contributing to GBM malignant progression by activating the PI3K-Akt signaling pathway. In translational medicine, we found that blocking WEE2-AS1 expression improved the therapeutic sensitivity of dasatinib, a central nervous system penetrant that is FDA-approved in GBM.

**Conclusions:** Overall, this work highlights that WEE2-AS1 may serve as a potential prognostic biomarker and therapeutic target in GBM, the knockdown of which significantly improves the efficacy of dasatinib, providing a promising strategy for improving targeted combination therapy for GBM patients.

## Introduction

Glioblastoma (GBM), which is highly aggressive, is considered one of the most devastating and fatal tumors. Despite advances in surgery and medicine, the prognosis of most GBM patients remains poor 1-4. Therefore, there is an urgent need to find and develop more efficient targeted therapeutic strategies to improve the prognosis of GBM patients.

Evidence accumulated over the past decade shows that long noncoding RNAs (lncRNAs), as a key regulators of gene expression, have a complex regulatory relationship with tumor-related gene expression levels at the epigenetic, transcriptional and posttranscriptional levels, which is closely related to tumorigenesis and malignant progression 5,6. The tissue-specific and condition-specific expression patterns of lncRNAs suggest that they are potential biomarkers and provide a theoretical basis for targeting them clinically 7,8. Therefore, a global depiction of lncRNA expression patterns in GBM tumor tissues can improve our understanding of GBM tissue-specific lncRNAs and provide a theoretical basis for the diagnosis and development of targeted therapeutic strategies for GBM patients.

N^6^-Methyladenosine (m^6^A) represents the most prevalent methylation modification of mRNAs and lncRNAs in eukaryotes 9-11, and it regulates almost every aspect of mRNA metabolism, including mRNA splicing 12, translation 13,14, stability 15 and decay 16, regulating gene expression at the posttranscriptional level. RNA m^6^A modification is catalyzed by the m^6^A methyltransferase complex (MTC), which is composed of methyltransferase-like 3 and 14 (METTL3 and METTL14, respectively) and their cofactors, Wilms tumor 1-associated protein (WTAP), KIAA1429 and RBM15, removed by m^6^A demethylases, such as FTO and ALKBH5, and detected by m^6^A readers, including YTHDF1/2/3, YTHDC1/2, IGF2BP1/2/3 and others, by interacting with distinct subsets of m^6^A sites to mediate different effects on mRNA metabolism, keeping m^6^A modification in a dynamic balance 17. Given the functional importance of RNA m^6^A modifications in normal biological processes, a growing number of validation studies suggest that dysregulation of RNA m^6^A modifications also contributes to GBM initiation, progression and drug resistance 18,19. However, the distribution and function of m^6^A modifications in lncRNAs, especially the role of elevated lncRNAs in GBM tissues, have not been fully revealed thus far. Understanding the mechanism of m^6^A-regulated lncRNAs in GBM pathogenesis will provide an important theoretical basis for clinical diagnosis and targeted therapy for GBM patients.

In this study, we reported the highly distinctive characteristics of m^6^A-modified lncRNAs in GBM tissues compared with normal brain tissues through m6A high-throughput sequencing (m^6^A-seq) for the first time. Through a systematic analysis, we identified a novel lncRNA, WEE2-AS1, which was significantly upregulated at both the m^6^A modification level and transcription level, and its high expression was correlated with a poorer prognosis in GBM. Functionally, we confirmed that WEE2-AS1 promoted GBM pathogenesis and malignant development in vitro and in vivo. Mechanistically, METTL3 mediated the m^6^A modification of WEE2-AS1 and enhanced its expression in an IGF2BP3-dependent manner. Moreover, WEE2-AS1 promoted RPN2 protein stabilization by preventing CUL2-mediated RPN2 K322 ubiquitination, thereby contributing to GBM malignant progression by activating the AKT signaling pathway. In translational medicine, we found that blocking WEE2-AS1 expression improved the therapeutic sensitivity of dasatinib, a CNS penetrant that is FDA-approved in GBM. Cumulatively, this work highlights that WEE2-AS1 may serve as a potential prognostic biomarker and therapeutic target in GBM, the knockdown of which significantly improved the efficacy of dasatinib in GBM, providing a promising strategy for improving targeted combination therapy for GBM patients.

## Results

### The m^6^A-modified lncRNA WEE2-AS1 is highly expressed in GBM

**Fig 1:**
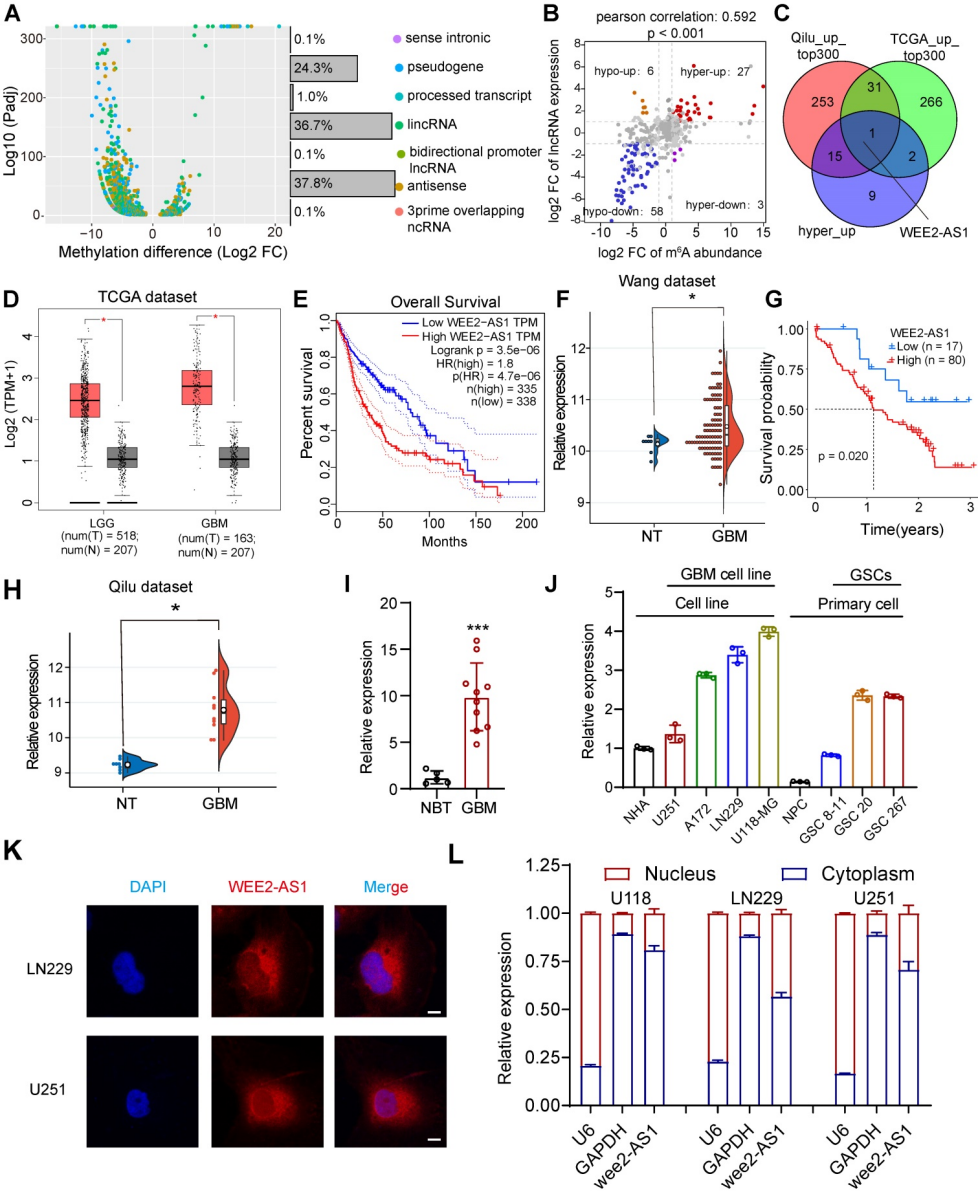
** Identification and characterization of the m6A-modified lncRNA WEE2-AS1 as a novel potential oncogene in GBM. A** Differentially methylated lncRNA types identified in three GBM tumor tissues compared with three normal brain tissues (NBTs) through m6A-seq. **B** Dot plot of Log2FC (lncRNA expression) versus Log2FC (differential m6A methylation) values showing a positive correlation between the overall m6A methylation level and the lncRNA expression level (Pearson's r = 0.592; P < 0.001) and the distribution of genes with significant changes in both the m6A (FC > 2, Padj < 0.05) and corresponding lncRNA expression levels in GBM samples compared with NBTs (FC > 2, Padj < 0.05). **C** Venn diagram showing the overlap of upregulated lncRNAs with the top 300 average expression levels in the TCGA dataset and the Qilu dataset, as well as lncRNAs in the hyper-up quadrant. **D** The GEPIA database showing that WEE2-AS1 was significantly overexpressed in glioma tissues compared with GTEx NBTs. **E** Kaplan-Meier survival curves showing that WEE2-AS1 is a prognostic risk factor in glioma. Log-rank analysis was used, P=3.5e-06. **F** WEE2-AS1 was significantly overexpressed in GBM tissues compared with NBTs in the Wang cohort. **G** Kaplan-Meier survival curves showing that WEE2-AS1 is a prognostic risk factor in GBM. Log-rank analysis was used, P=0.02. **H** WEE2-AS1 was significantly overexpressed in GBM tissues compared with NBTs in the Qilu cohort. qRT-PCR assays showing that the relative expression of WEE2-AS1 was significantly upregulated in (**I**) ten GBM tissues compared to five NBTs collected from the Qilu cohort and (**J**) in GBM cells, as well as GSCs, compared to their respective corresponding normal cells. Data are presented as the mean ± SD. **K** RNA-FISH assays showing the subcellular localization of WEE2-AS1 (Cy3) in LN229 and U251 GBM cells. Nuclei were stained with DAPI (blue). Scale bar, 5 μm. The statistical significance is shown as *P < 0.05; **P < 0.01; ***P < 0.001; ****P < 0.0001. **L** qRT-PCR assays showing the relative expression of WEE2-AS1 in the cytoplasmic (GAPDH was used as a cytoplasmic marker) and nuclear (U6 was used as a nuclear marker) fractions. Data are presented as the mean ± SD.

To understand the differences in the m^6^A modification patterns of lncRNAs in GBM, we performed m^6^A-seq assays on three human GBM tumor tissues and three normal brain tissues (NBTs). The results showed significant changes in the m^6^A modification abundance of a significant number of lncRNAs, among which pseudogene (24.3%), lincRNA (36.7%) and antisense (37.8%) lncRNAs accounted for most of them (Figure 1A, |fold change (FC)| > 2, Padj < 0.05). We then analyzed lncRNA expression profiles in 8 NBTs and 10 primary GBM tissues collected from Qilu Hospital and found that compared with NBTs, 1278 lncRNAs were differentially expressed in GBM tissues (|logFC| > 1 and false discovery rate (FDR) < 0.05), including 627 downregulated genes and 651 upregulated genes (Table S2-1). By conjoint analysis of m^6^A-seq and RNA-seq data, we discovered a positive correlation between differentially methylated m^6^A modification abundance and corresponding lncRNA expression levels in GBM tissues (Figure 1B, Pearson correlation: 0.592, P < 0.001), and the genes were mainly divided into four groups, including 27 hypermethylated and upregulated genes ('hyper-up'), 58 hypomethylated and downregulated genes ('hypo-down'), 3 hypermethylated but downregulated genes ('hyper-down') and 6 hypomethylated but upregulated genes ('hypo-up').

To identify potential oncogenic m^6^A-modified lncRNAs involved in the tumorigenesis and progression of GBM, we then analyzed lncRNA expression profiles in the TCGA GBM dataset and found that compared with NBTs, 2626 lncRNAs were differentially expressed in 153 primary GBM tissues (|logFC| > 2 and FDR < 0.05), including 921 downregulated genes and 1705 upregulated genes (Table S2-2). Subsequently, we intersected the lncRNAs with average expression in the top 300 of the TCGA dataset and the Qilu dataset with the lncRNAs in the hyper-up quadrant and identified WEE2-AS1, the only lncRNA with simultaneously upregulated m^6^A methylation abundance and expression levels (Figure 1C). The TCGA data confirmed that the expression of WEE2-AS1 was significantly higher in gliomas (LGG and GBM) than in normal tissues, with the highest expression in GBM (Figure 1D). Patients with high expression of WEE2-AS1 had a significantly poorer prognosis (Figure 1E), and the same results were obtained in another GBM dataset 20 (Figure 1F, G). WEE2-AS1 was also markedly upregulated in GBM tissues compared with NBTs in the Qilu cohort (Figure 1H). The in vitro qRT-PCR results further validated these results (Figure 1I). Additionally, we also detected the expression of WEE2-AS1 in both GBM and GSCs compared to their corresponding normal cells (Figure 1J), the results showed that WEE2-AS1 expression was upregulated in the GBM cell line compared to the normal human astrocyte (NHA) cell line. Meanwhile, WEE2-AS1 expression was upregulated in glioma stem cells compared with neural progenitor cells (NPCs). The functions of lncRNAs are mostly related to their intracellular localization. Nuclear-cytoplasmic fractionation experiments and FISH assays showed that WEE2-AS1 was mostly localized to the cytoplasm (Figure 1K-L). Taken together, these results suggested that WEE2-AS1 was significantly upregulated in GBM tissues and served as a prognostic risk factor, suggesting its possible involvement in GBM malignant progression.

### WEE2-AS1 promotes the proliferation and invasion of GBM cells in vitro and in vivo

To investigate the potential biological role of WEE2-AS1 in the progression of GBM, we performed KEGG enrichment analysis of genes that were significantly upregulated in the WEE2-AS1-high group. These genes were significantly enriched in many pathways involved in metabolism and human diseases and classical signaling pathways involved in tumorigenesis, such as the PI3K-Akt signaling pathway and ECM-receptor interaction (Figure S1A-B and Table S3).

To explore the function of WEE2-AS1 in GBM proliferation, metastasis and invasion, we sought to characterize the changes in cell biological behavior in WEE2-AS1-silenced and WEE2-AS1-overexpressing GBM cells in vitro. WEE2-AS1 was efficiently knocked down by two shRNAs (sh-WEE2-AS1-1 and sh-WEE2-AS1-2) in the U118MG and LN229 GBM cell lines and in GSC20 GSCs with higher expression (Figure 1J and Figure S2A) and overexpressed in the A172 and U251 GBM cell lines and GSC267 GSCs with lower expression (Figure S2B).

**Fig 2:**
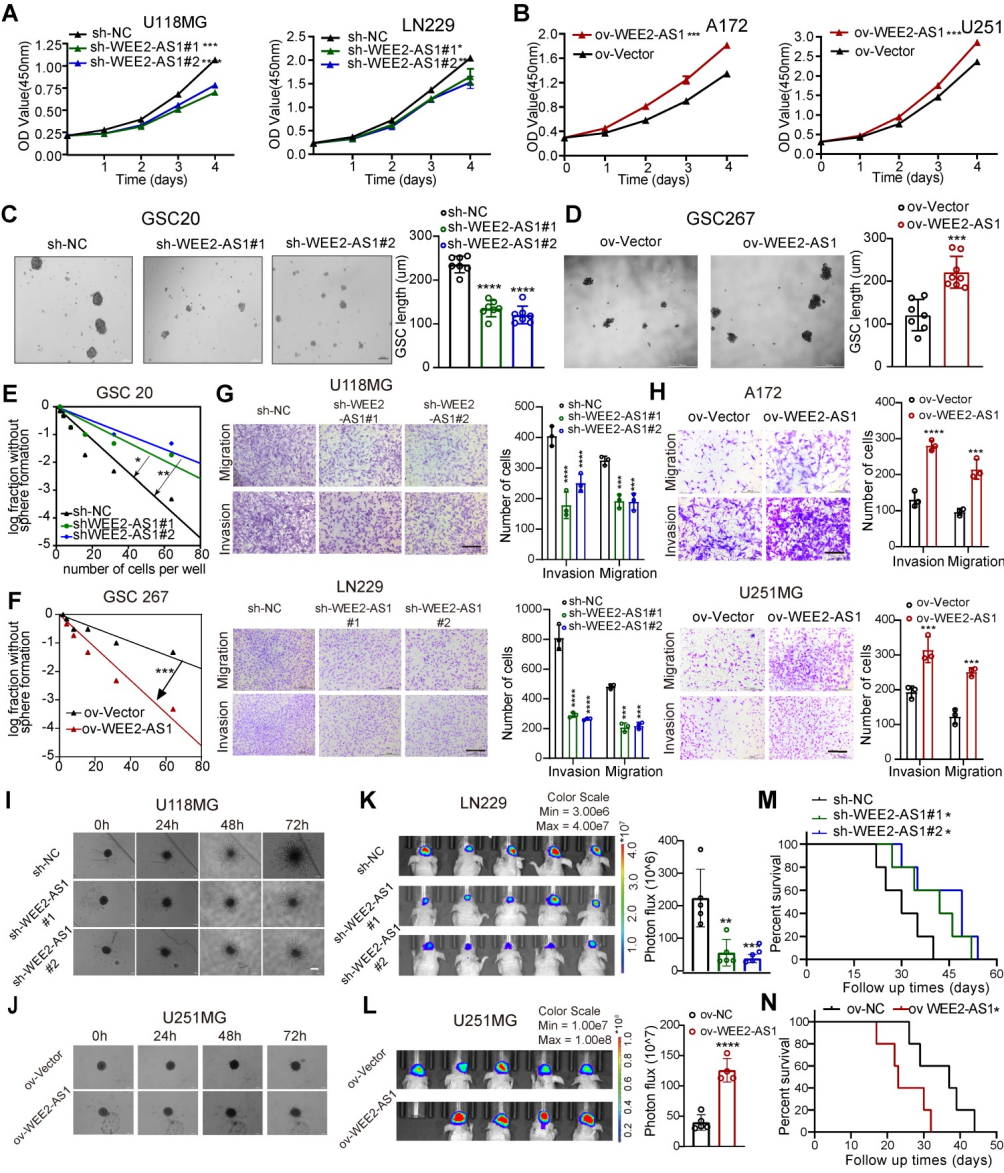
** WEE2-AS1 promotes the proliferation, migration and invasion of GBM cells in vitro and in vivo.** CCK-8 assays showing the proliferation ability of GBM cells transfected with (**A**) sh-NC or sh-WEE2-AS1 and (**B**) ov-NC or ov-WEE2-AS1, n = 3. Representative tumor sphere formation images of GSCs transfected with (**C**) sh-NC or sh-WEE2-AS1 and (**D**) ov-NC or ov-WEE2-AS1; scale bar, 200 µm. The quantification histogram represents the average sphere diameter. Data represent the mean ± SD from at least three independent experiments. Limiting dilution assays for GSCs transfected with (**E**) sh-NC or sh-WEE2-As1 and (**F**) ov-NC or ov-WEE2-AS1. Representative Transwell migration and invasion assays showing the migration and invasion ability of GBM cells transfected with (**G**) sh-NC or sh-WEE2-AS1 and (**H**) ov-NC or ov-WEE2-AS1; scale bar, 200 µm. The quantification histogram represents the relative cell numbers. Data represent the mean ± SD from at least three independent experiments. 3D tumor spheroid invasion assays showing the invasion ability of GBM cells transfected with (**I**) sh-NC or sh-WEE2-AS1 and (**J**) ov-NC or ov-WEE2-AS1. Bioluminescence image showing the tumor size of mice implanted with luciferase-labeled (**K**) LN229 cells expressing sh-WEE2-AS1 or sh-NC and (**L**) U251MG cells expressing ov-WEE2-AS1 or ov-NC at the indicated times. The quantification histogram represents the bioluminescent flux. Data represent the mean ± SD, n ≥ 4 for each group. Kaplan-Meier survival curves for mice implanted with luciferase-labeled (**M**) LN229 cells expressing sh-WEE2-AS1 or sh-NC and (**N**) U251MG cells expressing ov-WEE2-AS1 or ov-NC. Log-rank analysis was used, n ≥ 4 for each group. The statistical significance is shown as *P < 0.05; **P < 0.01; ***P < 0.001; ****P < 0.0001.

Knockdown of WEE2-AS1 significantly inhibited the proliferation of U118MG and LN229 GBM cells (Figure 2A and Figure S2C-D), while overexpression of WEE2-AS1 remarkably promoted these cellular behaviors in A172 and U251 GBM cells (Figure 2B and Figure S2E-F), as detected by CCK-8, colony formation and EdU assays. More importantly, we examined whether WEE2-AS1 was associated with the cell cycle by flow cytometry and found that WEE2-AS1 silencing induced G2/M arrest (Figure S2G-H). The Western blot analysis results also showed that knockdown of WEE2-AS1 increased the expression of p-CDK1 and P21 but decreased the expression of Cyclin B1, which is involved in G2/M phase progression (Figure S2I-J). Furthermore, we performed neurosphere formation and limiting dilution assays on GSC20 and GSC267 GSCs, which showed that WEE2-AS1 significantly promoted the tumorsphere expansion (Figure 2C-D) and sphere formation ability of GSCs (Figure 2E-F). In addition, Transwell and 3D collagen spheroid invasion assays showed that knockdown of WEE2-AS1 impaired the migration and invasion abilities of U118MG and LN229 GBM cells, while overexpression of WEE2-AS1 remarkably promoted these cellular behaviors (Figure 2G-J).

In addition, in vivo experiments showed that downregulation of WEE2-AS1 significantly inhibited tumor growth and prolonged the survival time of tumor-bearing mice, while overexpression of WEE2-AS1 had the opposite results (Figure 2K-N). HE staining of excised tumor sections showed lower invasive capacity in tumor tissues with knockdown of WEE2-AS1 than those in the vector group, while the opposite result was observed in tumor tissues overexpressing WEE2-AS1 (Figure S3A-B). In addition, immunohistochemistry (IHC) analysis indicated that the expression of Ki67 (a proliferation marker) and N-cadherin (an invasiveness marker) in WEE2-AS1-knockdown tumor tissues was lower than that in the vector group, while WEE2-AS1 overexpression showed the opposite results (Figure S3C-D). Collectively, these results suggested that WEE2-AS1 exerted an oncogenic role in GBM by regulating cell proliferation, migration and invasion.

### METTL3-mediated m^6^A modification enhances WEE2-AS1 stabilization in an IGF2BP3-dependent manner

**Fig 3:**
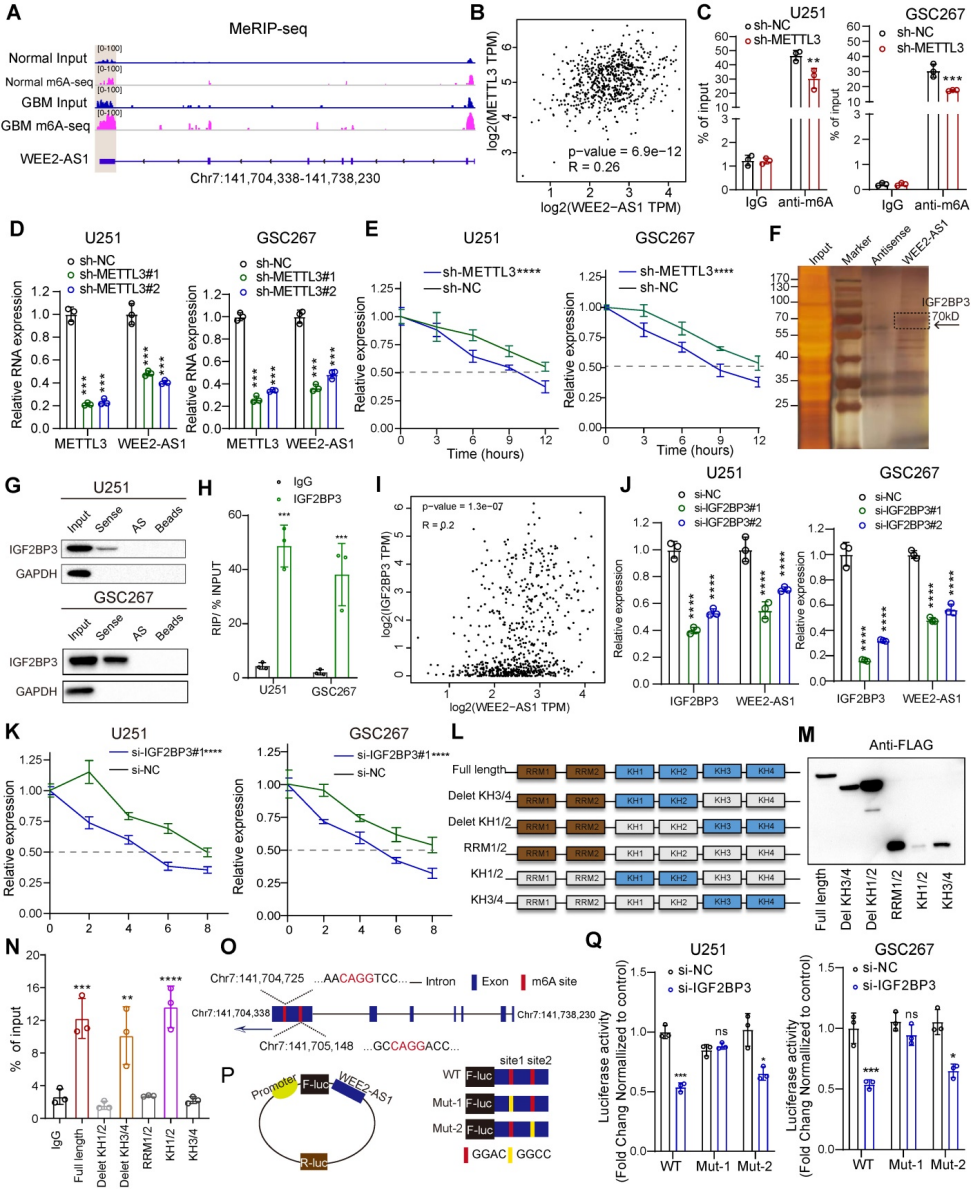
** METTL3-mediated m6A modification enhanced WEE2-AS1 stabilization in an IGF2BP3-dependent manner. A** IGV software showing the m6A peak distribution on WEE2-AS1 in GBM tissues and NBTs. **B** The correlation between the m6A methylesterase METTL3 and WEE2-AS1 in glioma tissues from the GEPIA database. **C** MeRIP assay showing that WEE2-AS1 was highly enriched by the m6A antibody, and the enrichment was downregulated in METTL3-knockdown (left) U251 and (right) GSC267 cells. Data represent the mean ± SD from at least three independent experiments. **D** qRT-PCR assays showing the relative expression of METTL3 and WEE2-AS1 in (left) U251MG GBM cells and (right) GSC267 GSCs transfected with sh-NC or sh-METTL3. Data represent the mean ± SD from at least three independent experiments. **E** The RNA stability of WEE2-AS1 was measured by qRT-PCR in METTL3-knockdown (left) U251MG and (right) GSC267 cells treated with Act-D for the indicated times. **F** Silver staining assays showing the proteins that interacted with WEE2-AS1, which were identified by RNA pulldown/mass spectrometry. **G** Western blot analysis showing the interaction between WEE2-AS1 and IGF2BP3 in U251 and GSC267 cells. WEE2-AS1 antisense beads and beads served as negative controls. **H** RIP-qPCR assays showing the relative enrichment of WEE2-AS1 detected by IGF2BP3 antibody. Data represent the mean ± SD from at least three independent experiments. **I** The correlation between the m6A reader IGF2BP3 and WEE2-AS1 in glioma tissues from the GEPIA database. **J** qRT-PCR assays showing the relative expression of IGF2BP3 and WEE2-AS1 in (left) U251MG GBM cells and (right) GSC267 GSCs transfected with sh-NC or sh-METTL3. Data represent the mean ± SD from at least three independent experiments. **K** The RNA stability of WEE2-AS1 was measured by qRT-PCR in IGF2BP3-knockdown (left) U251MG and (right) GSC267 cells treated with Act-D for the indicated times.** L** Schematic structures showing the Flag-tagged vectors of RRM and KH domains of IGF2BP3.** M** Western blot showing the FLAG-tagged domains of IGF2BP3 in HEK293T cells. **N** RIP-qPCR analysis for WEE2-AS1 enrichment in HEK293T cells transfected with FLAG-tagged full-length and FLAG-tagged truncated plasmids. Data represent the mean ± SD from at least three independent experiments. **O** Schematic representation of the positions of m6A motifs in exon 7 of WEE2-AS1. **P** Schematic representation of the mutated (GGAC to GGCC) firefly luciferase reporter vector. **Q** Luciferase assays showing the main locus of m6A-mediated WEE2-AS1 in U251MG and GSC267 cells. The statistical significance is shown as follows: ns>0.05, **P < 0.05; **P < 0.01; ***P < 0.001; ****P < 0.0001.

M^6^A modifications regulate all phases of the RNA life cycle, such as RNA splicing, stabilization, degradation and nuclear export 9,21, thereby regulating RNA expression and function. Since our joint analysis of m^6^A-seq and RNA-seq data revealed that both the abundance of m^6^A modifications and gene expression of WEE2-AS1 were significantly upregulated in GBM tissues compared with normal tissues, we next explored the underlying mechanism of m^6^A-mediated WEE2-AS1 expression. Visualization of m^6^A-seq using IGV software revealed that compared with NBTs, the abundance of m^6^A modifications on exon 7 of WEE2-AS1 in GBM tissues was upregulated (Figure 3A). GEPIA and Wang database analysis showed a significant positive correlation between the expression of the m6A methyltransferases METTL3 and WEE2-AS1 in glioma tissues (Figure 3B, Figure S3E). Furthermore, MeRIP-qPCR assays demonstrated that WEE2-AS1 could be significantly enriched by an m^6^A antibody, enrichment of which was significantly downregulated in METTL3-knockdown GBM cells compared with the NC group (Figure 3C and Figure S4A). Moreover, knockdown of METTL3 significantly reduced the expression level of WEE2-AS1 in GBM cells (Figure 3D). Thus, we proposed that m^6^A might affect the stability of WEE-AS1. To verify this speculation, we evaluated the effect of METTL3 on the stability of WEE2-AS1 via actinomycin D RNA stability experiments and found that the half-life of WEE2-AS1 was significantly shorter in the METTL3-knockdown group than in the NC group (Figure 3E). Our subsequent RNA pulldown/mass spectrometry results revealed that WEE2-AS1 bound to the m^6^A reader IGF2BP3, a well-known RNA-binding protein belonging to the insulin-like growth factor 2 mRNA-binding protein (IGF2BP) family, to stabilize their target RNA (Figure 3F and Table S4-1), which has been reported to play oncogenic roles in GBM progression 22. Both RNA pulldown and RIP-qPCR assays verified that WEE2-AS1 could bind to the IGF2BP3 protein (Figure 3G-H). GEPIA and Wang database analysis also showed a significant positive correlation between the expression of IGF2BP3 and WEE2-AS1 in glioma tissues (Figure 3I, Figure S3E). Consistent with the METTL3 results, knockdown of IGF2BP3 significantly reduced the expression level and stability of WEE2-AS1 (Figure 3J-K and Figure S4B). IGF2BP3 consists of 2 RNA recognition motifs (RRMs) and 4 K homology structural domains (KHs). Thus, we then established 6 FLAG-tagged vectors and detected which structural domains interacted with WEE2-AS1 (Figure 3L-M). RIP-PCR assays confirmed that the KH 1-2 domains were essential for the recognition of WEE2-AS1 (Figure 3N).

To further elucidate the mechanism of m^6^A regulation of WEE2-AS1 expression, we explored its m^6^A modification sites. Our m^6^A-seq data showed that m^6^A peaks in exon 7 regions ranged from chr7:141,704,338 to Ch7:141,705,757, where two m^6^A motifs (GGACs) were identified (Figure 3O). We then used a luciferase reporter containing firefly luciferase, followed by wild-type WEE2-AS1, mutant 1 (Mut-1) or mutant 2 (Mut-2), where putative m^6^A sites were mutated (GGAC to GGCC) in exon 7 (Figure 3P). As shown in Figure 3Q, the luciferase activity of the wild-type and Mut-2 WEE2-AS-fused reporter was significantly reduced in the presence of IGF2BP3 knockdown, but the Mut-1 WEE2-AS1-fused reporter showed no significant difference (Figure 3Q), indicating that site 1 in exon 7 of WEE2-AS1 was the main locus of m^6^A regulation. Taken together, our results suggested that METTL3-mediated m^6^A modification of WEE2-AS1 promoted its expression by enhancing its stability in an IGF2BP3-dependent manner.

### WEE2-AS1 acts as a scaffold for RPN2 and contributes to GBM malignant progression by activating the AKT signaling pathway

**Fig 4:**
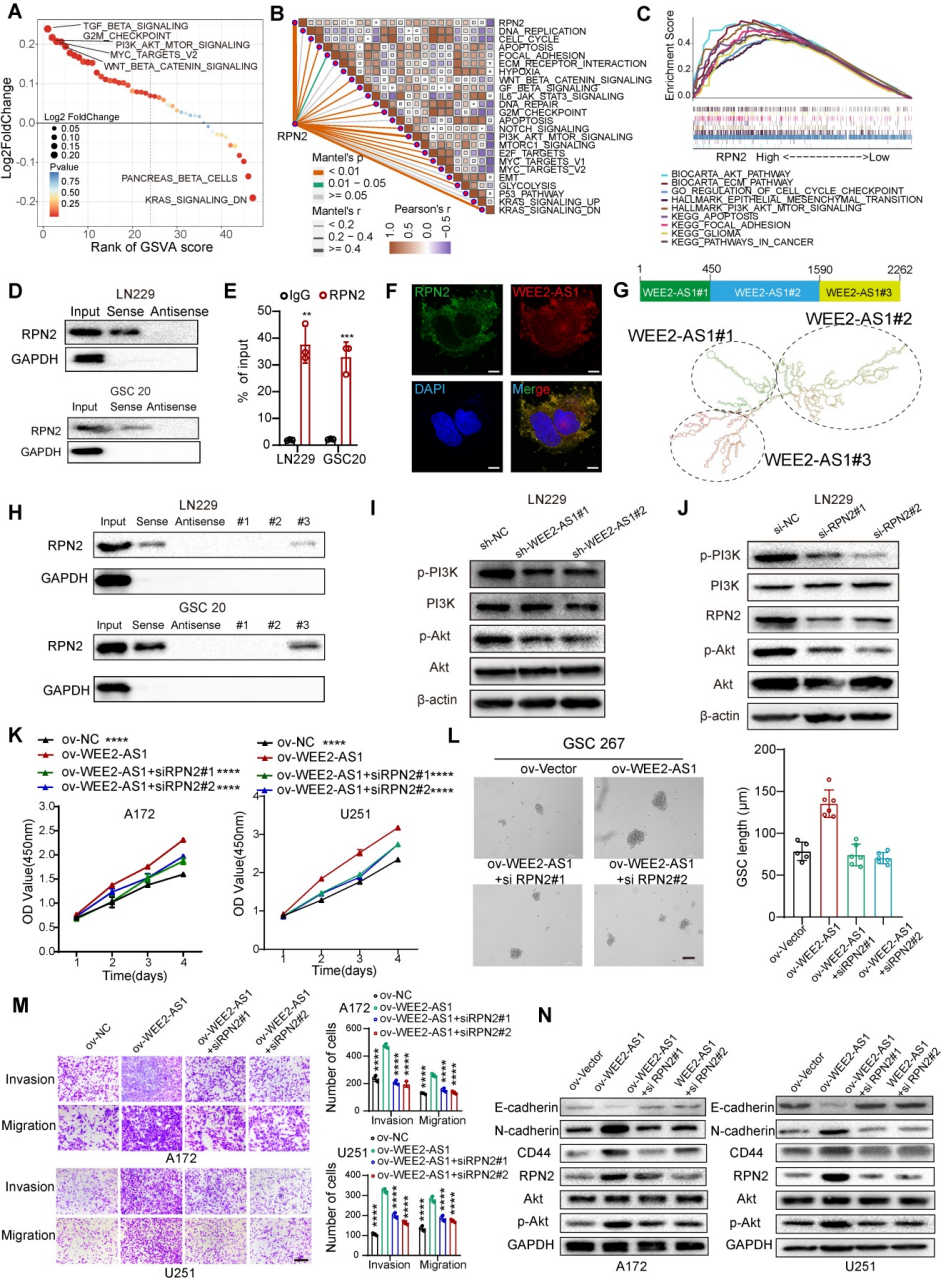
** WEE2-AS1 interacts with RPN2 protein and contributes to GBM malignant progression by activating the PI3K-AKT signaling pathway. A** GSVA showing differences in hallmark biological pathways between the high and low RPN2 samples in the TCGA GBM cohort. Scatter plots were used to visualize these differences in pathways. The size of the circle indicates the size of the fold change (FC), and the color indicates the statistical significance of the difference. The red color indicates statistical significance, and blue indicates statistical insignificance. **B** Correlations between RPN2 and the enrichment scores of cancer hallmark pathways in the TCGA GBM cohort. **C** GSEA showing that classical carcinogenic pathways involved in tumor pathogenesis were significantly enriched in the high RPN2 expression group in the TCGA GBM dataset. **D** Western blot analysis showing the interaction between WEE2-AS1 and RPN2 in (top) LN229 GBM cells and (bottom) GSC20 GSCs. **E** RIP-qPCR assays showing the relative enrichment of WEE2-AS1 detected by RPN2 antibody. Data represent the mean ± SD from at least three independent experiments. **F** RNA FISH-immunofluorescence (FISH-IF) assays showing the colocalization of WEE2-AS1 and RPN2 in GBM cells. **G** Secondary structure of WEE2-AS1 predicted by the RNAfold WebServer. **H** RNA pull-down assay by in vitro transcribed biotinylated RNAs corresponding to different fragments of WEE2-AS1 in LN229 and GSC20 cells. Western blot assays showing the phosphorylation levels of AKT in LN229 GBM cells transfected with (**I**) sh-NC or sh-WEE2-AS and (**J**) si-NC or si-RPN2. **K** CCK-8 assays showing the proliferation ability of GBM cells cotransfected with ov-NC or ov-WEE2-AS1 and siRPN2 as indicated. **L** Representative tumor sphere formation images of GSCs cotransfected with ov-NC or ov-WEE2-AS1 and si-RPN2 as indicated; scale bar, 200 µm. The quantification histogram represents the average sphere diameter. Data represent the mean ± SD from at least three independent experiments. **M** Representative Transwell migration and invasion assays showing the migration and invasion ability of GBM cells cotransfected with ov-NC or ov-WEE2-AS1 and siRPN2 as indicated; scale bar, 200 µm. The quantification histogram represents the relative cell numbers. Data represent the mean ± SD from at least three independent experiments. **N** Western blot assays showing E-cadherin, N-cadherin, CD44, RPN2, AKT and p-AKT protein levels in GBM cells cotransfected with ov-NC or ov-WEE2-AS1 and siRPN2 as indicated. The statistical significance is shown as *P < 0.05; **P < 0.01; ***P < 0.001; ****P < 0.0001.

Numerous studies have shown that cytoplasm-retained lncRNAs can interact with proteins to participate in cellular regulation 5. Subsequently, we then screened the remaining WEE2-AS1-labeled pulldown proteins, and found 48 RBPs 23, excluding IGF2BP3, which were potential target proteins interacting with WEE2-AS1 (Figure S4C and Table S4-2). Univariate Cox regression analysis was performed and showed that RPN2 and PMSD1 were significant survival-related risk genes (Table S4-3). The Human Protein Atlas (HPA) database revealed that RPN2 was mainly distributed in the cytoplasm, while PMSD1 was mainly distributed in the nucleus (Figure S4D). Furthermore, the GEPIA database showed that RPN2 was significantly upregulated in TCGA GBM samples compared with GTEx NBTs, while the difference in PMSD1 expression was not significant (Figure S4E). Therefore, we next focused only on the biological function of the RPN2 protein in GBM. The Kaplan-Meier survival analysis results from multiple GBM databases also validated RPN2 as a prognostic risk factor (Figure S4F). In addition, the cancer-promoting signaling pathways were significantly upregulated in RPN2-high GBM samples compared with RPN2-low GBM samples (Figure 4A), as estimated via the GSVA algorithm. We also analyzed the correlation between RPN2 and cancer hallmark pathways. As shown in Figure 4B, there was a significant positive correlation between RPN2 and these cancer-promoting pathways. Moreover, similar to GSVA enrichment analysis, the GSEA results also showed that classical pathways involved in tumor pathogenesis were significantly enriched in the high RPN2 expression group in the TCGA GBM dataset (Figure 4C). Thus, we speculated that WEE2-AS1 might promote the malignant progression of GBM by influencing the function of RPN2. Next, we performed RNA pull-down and RIP-qPCR assays and validated the interaction between WEE2-AS1 and RPN2 (Figure 4D-E). RNA FISH-immunofluorescence (FISH-IF) analysis was also performed and showed that WEE2-AS1 colocalized with RPN2 in the cytoplasm (Figure 4F). To further investigate the secondary structure of WEE2-AS1 interacting with RPN2, the RNAfold WebServer was used to predict the structure of the WEE2-AS1 molecule and divide it into three major sub-structure, each containing a base-pairing structure and a hairpin structure (Figure 4G). RNA pull-down results showed that WEE2-AS1#3 bound to RPN2 as efficiently as full-length WEE2-AS1, and other sub-structure lost their binding capacity (Figure 4H), indicating that nucleotides 1590-2262 were required for the association with RPN2. Next, we explored the potential biological pathways downstream of the WEE2-AS/RPN2 axis. Functional enrichment analysis showed that GBM samples with high expression of both WEE2-AS1 and RPN2 were significantly enriched in the AKT signaling pathway (Figure 4A-C and Figure S1). In addition, PI3K-Akt was a downstream signaling pathway of RPN2 in other tumors articles 24,25. Thus, we then investigated the phosphorylation levels of PI3K and Akt in WEE2-AS1 knockdown and overexpression GBM. As shown in Figure 4I and Figure S4G, overexpression of WEE2-AS1 enhanced the phosphorylation level of Akt, but WEE2-AS1 knockdown inhibited the phosphorylation level of Akt. Consistent with the results obtained for WEE2-AS1, knockdown of RPN2 also inhibited activation of the AKT signaling pathway (Figure 4J and Figure S4H). Rescue assays showed that RPN2 knockdown compensated for the increased proliferation, invasion and migration capacity of GBM cells and self-renewal ability of GSCs, as well as activated AKT signaling pathway, caused by exogenous overexpression of WEE2-AS1 (Figure 4K-N and Figure S4I). *In vivo* rescue experiments also confirmed these findings (Figure S5). Taken together, these results demonstrated that the lncRNA WEE2-AS1 can act as a scaffold for RPN2 and the WEE2-AS1/RPN2 complex activating the downstream PI3K-AKT signaling pathways to promote malignant progression of GBM.

### WEE2-AS1 stabilizes RPN2 protein by preventing CUL2-mediated ubiquitin-proteasome degradation

**Fig 5:**
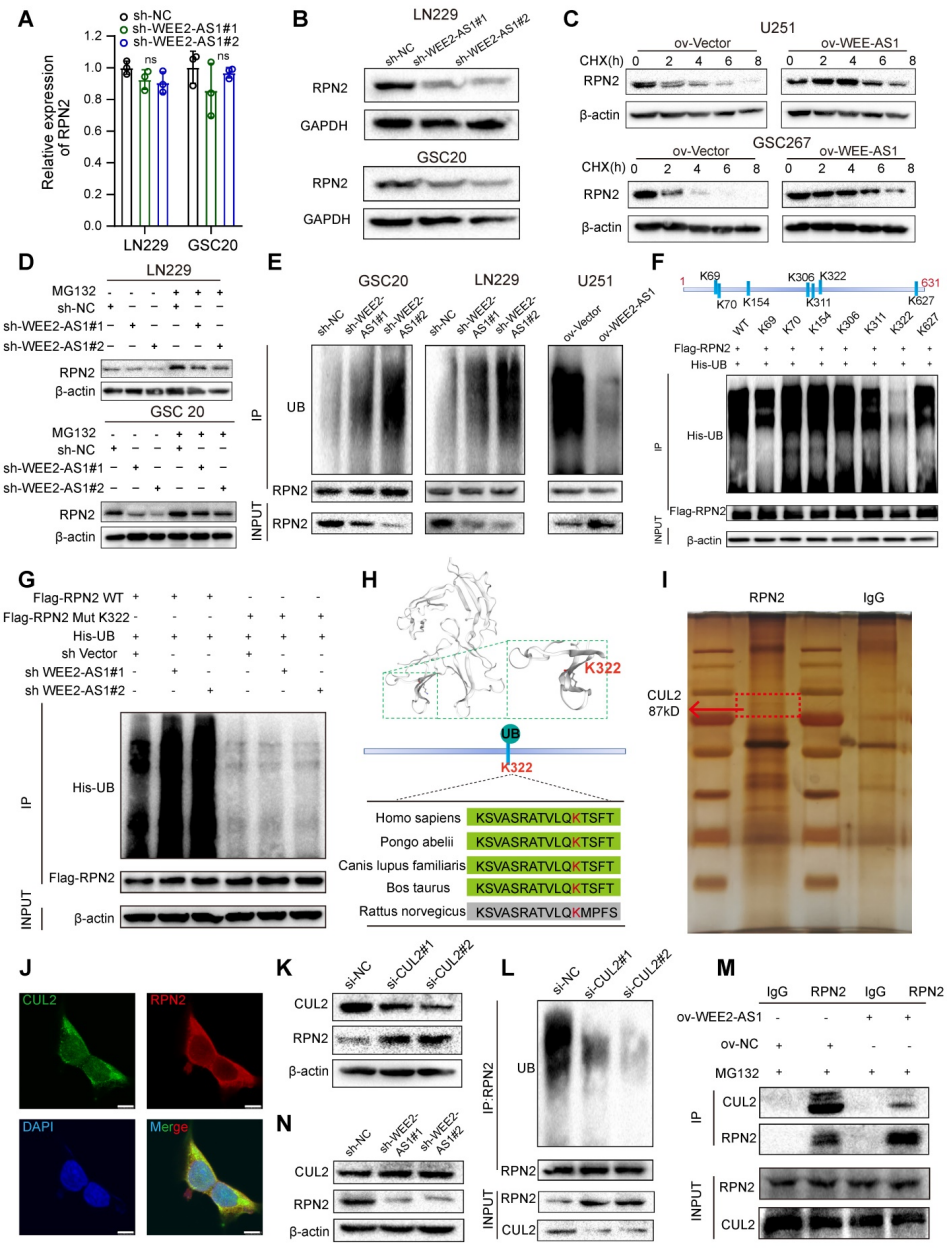
** WEE2-AS1 stabilizes RPN2 protein by preventing CUL2-mediated ubiquitin-proteasome degradation. A** qRT-PCR assays showing the relative expression in GBM cells transfected with sh-NC or sh-WEE2-AS1. Data represent the mean ± SD from at least three independent experiments. **B** Western blot assays showing RPN2 protein levels in GBM cells transfected with sh-NC or sh-WEE2-AS1. Western blot assays showing RPN2 protein level in GBM cells (**C**) transfected with ov-NC or ov-WEE2-AS1, treated with the protein synthesis inhibitor cycloheximide (CHX, 25 μg/mL) at the indicated time, and (**D**) transfected with sh-NC or sh-WEE2-AS1, treated with the proteasome inhibitor MG132 (5 μmol/L) at the indicated time. **E** Co-IP assays showing the RPN2 ubiquitination levels in GBM cells transfected with sh-NC or sh-WEE2-AS1. UB, ubiquitination. **F** Top, the potential ubiquitination sites of RPN2 predicted via BDM-PUM (http://bdmpub.biocuckoo.org/) and the UbiBrowser database (http://ubibrowser.ncpsb.org/). Bottom, Co-IP assays showing the RPN2 ubiquitination level after transfection with Flag-tagged wild-type or mutant RPN2 KR vectors. KR, mutation of lysine (K) to arginine **G** Co-IP assays showing the RPN2 ubiquitination level in GBM cells co-transfected with sh-NC or sh-WEE2-AS1 and Flag-tagged wild-type or K322 mutant RPN2 KR vectors. **H** Top, Crystal structure of RPN2 proteins with K322. Bottom, conservation ability of the K322 ub site on the RPN2 protein. **I** Silver staining assays showing the proteins that interacted with RPN2, which were identified by co-IP/mass spectrometry; arrows indicate CUL2 protein bands. **J** Immunofluorescence staining experiments showing the colocalization of RPN2 and CUL2 in GBM cells. Scale bar, 25 μm. **K** Western blot assays showing CUL2 and RPN2 protein levels in GBM cells transfected with si-NC or si-CUL2. **L** Co-IP assays showing the RPN2 ubiquitination levels in GBM cells transfected with si-NC or si-CUL2. **M** Co-IP assays showing the intensity of the interaction between RPN2 and CUL2 in GBM cells transfected with ov-NC or ov-WEE2-AS1 and treated with MG132 at the indicated times. The statistical significance is shown as follows: ns>0.05. **N** Western blot assays showing CUL2 and RPN2 protein levels in GBM cells transfected with sh-NC or sh-WEE2-AS1.

Next, we explored the potential mechanism by which WEE2-AS1 interacted with the RPN2 protein. Our investigation indicated that WEE2-AS1 did not significantly alter the mRNA expression of RPN2 (Figure 5A), but promoted its protein level (Figure 5B) in GBM cells. We further observed that compared with the NC group, overexpression of WEE2-AS1 prolonged the half-life of RPN2 protein by blocking the degradation of RPN2 protein (Figure 5C), suggesting that WEE2-AS1 enhanced the stability of RPN2 to promote its protein level. The ubiquitin-proteasome system (UPS) is the main pathway of intracellular protein degradation 26. Our further investigation showed that WEE2-AS1 knockdown reduced the protein level of RPN2, which could be restored by the proteasome inhibitor MG132 (Figure 5D). Moreover, ubiquitination of RPN2 was elevated in WEE2-AS1-knockdown GBM cells and decreased in WEE2-AS1-overexpressing GBM cells compared with their corresponding NC groups (Figure 5E), indicating that WEE2-AS1 regulates the stability of RPN2 protein through ubiquitin-proteasome activity. Next, we used BDM-PUM (http://bdmpub.biocuckoo.org/) and the UbiBrowser database (http://ubibrowser.ncpsb.org/) to predict the ubiquitination sites of RPN2 and found seven potential ubiquitination sites (Figure 5F). Then, we mutated the potential ubiquitination site from lysine (K) to arginine (R) to inhibit ubiquitination. The IP results showed that compared with other groups, mutation of the K322 site greatly reduced RPN2 ubiquitination (Figure 5F), and the reduced ubiquitination level of RPN2 protein caused by knockdown of WEE2-AS1 disappeared after mutation at the K322 site (Figure 5G), highlighting K322 as the major ubiquitination site of RPN2. Furthermore, we predicted the structure of RPN2 through the Swiss Model Online website (https://swissmodel.expasy.org/) and visualized the K322 ubiquitination site, which is highly conserved among mammals (Figure 5H).

Ubiquitin ligase (E3) is one of the most critical and heterogeneous enzymes in the ubiquitination pathway 27, and there are no reports of E3 ubiquitin ligase-mediated degradation of RPN2 ubiquitination. To identify the E3 ligases involved in the proteasome-mediated degradation of RPN2, we performed co-IP experiments and mass spectrometry analysis and found that CUL2, a protective factor for GBM prognosis 28, that forms cullin-RING complexes to promote substrate ubiquitination and degradation 29, can bind to RPN2 (Figure 5I and Table S5). IF staining experiments confirmed the colocalization of RPN2 and CUL2 in GBM cells (Figure 5J). Furthermore, we found that compared with the NC group, the protein level of RPN2 was dramatically increased (Figure 5K), while its level of ubiquitination was obviously decreased in CUL2-knockdown GBM cells (Figure 5L). Functional rescue experiments also showed that CUL2 knockdown increased the proliferation, migration and invasion abilities of GBM cells, which could be reversed by RPN2 downregulation (Figure S4J- K), indicating that CUL2 could act as a ubiquitin ligase of RPN2 to regulate the malignant progression of GBM. Co-IP assays revealed that compared with the NC group, the binding intensity of CUL2 to RPN2 was obviously reduced in WEE2-AS1-overexpressing GBM cells (Figure 5M), while WEE2-AS1-knockdown did not affect CUL2 protein levels (Figure 5N), suggesting that WEE2-AS1 enhances the stability of RPN2 by inhibiting its binding to CUL2. Taken together, these results suggested that WEE2-AS1 stabilized RPN2 by preventing CUL2-mediated RPN2 K322 ubiquitination, thereby promoting the malignant progression of GBM.

### Knockdown of WEE2-AS1 improves the efficacy of dasatinib in GBM

**Fig 6:**
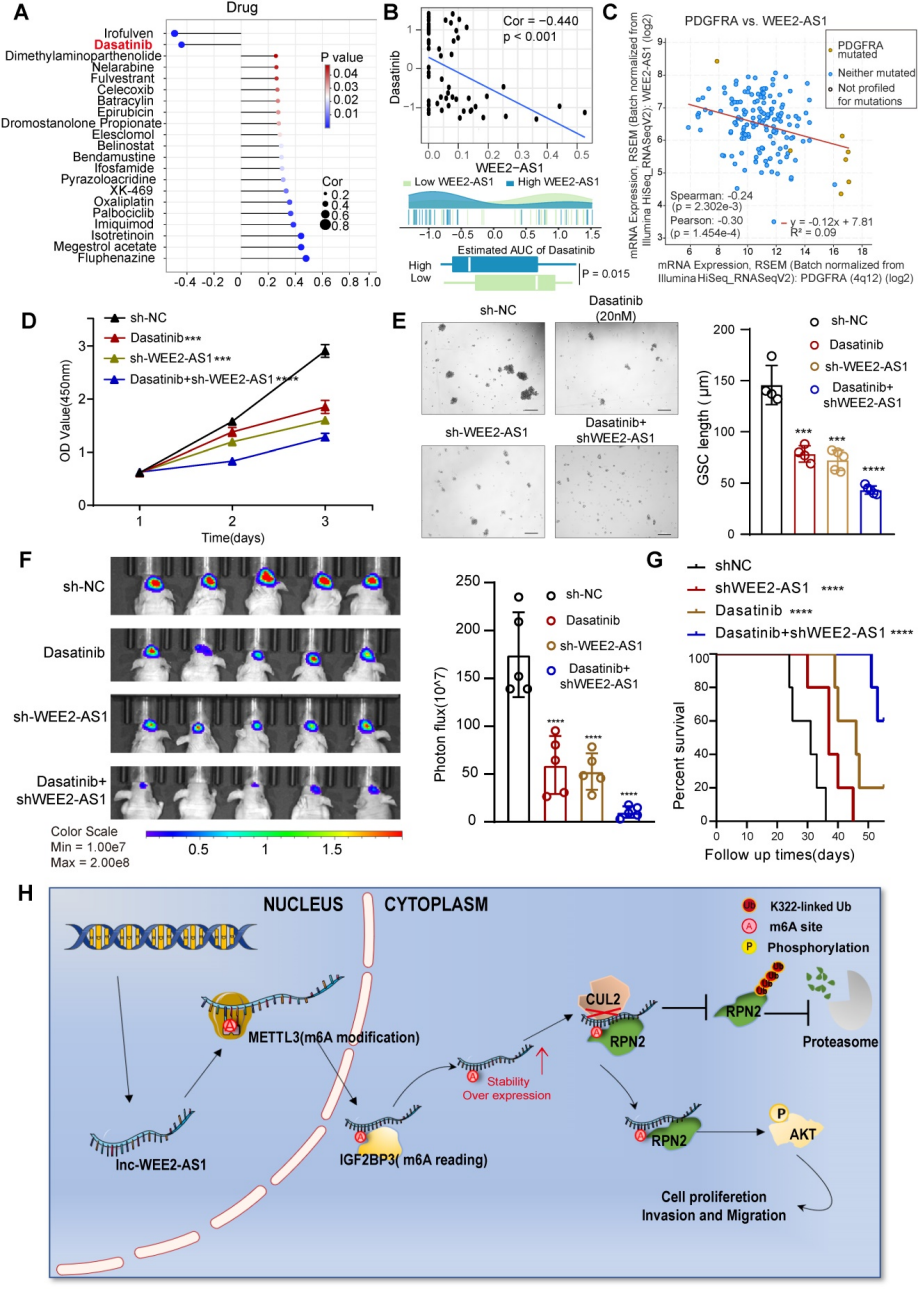
** Knockdown of WEE2-AS1 improves the efficacy of dasatinib in GBM. A** Spearman's correlation analysis between WEE2-AS1 and the bioavailability (AUC) of the drugs that have undergone clinical trials or received FDA approval was collected from the CellMiner database (https://discover.nci.nih.gov/cellminer/). **B** Top, Spearman's correlation between WEE2-AS1 and the AUC of dasatinib. Bottom, comparison of the estimated dasatinib AUC between the WEE2-AS1 high group and the low group. **C** Pearson correlation between the expression of WEE2-AS1 and PDGFRA. **D** CCK-8 assays showing the proliferation ability of GBM cells transfected with sh-NC or sh-WEE2-AS1 and treated with dasatinib (20 nM) for the indicated times. **E** Representative tumor sphere formation images of GSCs transfected with sh-NC or sh-WEE2-AS1 and treated with dasatinib (20 nM) for the indicated times. Scale bar, 200 μm. The quantification histogram represents the average sphere diameter. Data represent the mean ± SD from at least three independent experiments. **F** Bioluminescent image showing the tumor size of mice implanted with luciferase-labeled LN229 cells expressing sh-WEE2-AS1 or sh-NC and treated with dasatinib (10 mg/kg) for the indicated times. The quantification histogram represents the bioluminescent flux. Data represent the mean ± SD, n = 5 for each group. **G** Kaplan-Meier survival curves for mice implanted with luciferase-labeled LN229 cells expressing sh-WEE2-AS1 or sh-NC and treated with dasatinib (10 mg/kg). Log-rank analysis was used, n = 5 for each group. **H** Proposed working model of the functions of WEE2-AS1 in the malignant progression of GBM. The statistical significance is shown as ***P < 0.001 and ****P < 0.0001.

To further understand the effects of WEE2-AS1 on the drug response, we assessed the association between WEE2-AS1 and the response to drugs collected from the CellMiner database (https://discover.nci.nih.gov/cellminer/) that have undergone clinical trials or received FDA approval. Using Spearman's correlation analysis, we identified 21 drugs significantly associated with WEE2-AS1 (Figure 6A and Table S6), two of which (irofulven and dasatinib) were significantly negatively correlated with WEE2-AS1, implying that WEE2-AS1 might resist the therapeutic sensitivity of these two drugs. Dasatinib, a highly potent second-generation adenosine triphosphate-competitive inhibitor shown to be effective against multiple protein tyrosine kinases, including platelet-derived growth factor receptor (PDGFR) and the Src family of kinases 30, has been approved for pediatric chronic myeloid leukemia by the FDA 31. Intriguingly, given its intrinsic qualities (i.e., lipophilicity, size, and protein binding), dasatinib could cross the blood-brain barrier 32, emphasizing its potential as a CNS penetrant. Several studies have demonstrated improved efficacy of dasatinib in the treatment of GBM, especially in GBM carrying the PDGFRA mutation 33-36. Recently, scRNA-seq data for dasatinib-resistant GBM demonstrated increased AKT activation 37. Similarly, GBM cells with high expression of WEE2-AS1 also exhibited activation of the AKT signaling pathway. We found that compared to the low WEE2-AS1 expression group, the bioavailability of dasatinib was significantly lower in the high WEE2-AS1 expression group (Figure 6B). Further analysis revealed a significant negative correlation between WEE2-AS1 and PDGFRA expression (Figure 6C), suggesting that knockdown of WEE2-AS1 might enhance the sensitivity of GBM cells to dasatinib. Further functional experiments showed that compared with the control group, knockdown of WEE2-AS1 in combination with dasatinib significantly inhibited GBM cell proliferative capacity and GSC self-renewal ability in vitro (Figure 6D-E). In addition, in vivo experiments showed that downregulation of WEE2-AS1 in combination with dasatinib significantly inhibited tumor growth and prolonged the survival time of tumor-bearing mice (Figure 6F-G). Overall, our data demonstrated that blocking the expression of WEE2-AS1 might enhance the therapeutic efficacy of dasatinib in GBM.

## Discussion

M^6^A is a key RNA modification that plays an irreplaceable role in the pathogenesis of GBM. LncRNAs, as key regulators of gene expression, also play an important role in m^6^A modification expression and functional regulation. Therefore, global characterization of the specific expression of m^6^A-modified lncRNAs in GBM tissues can help elucidate the functions of m^6^A and lncRNAs in the development of GBM. Here, we identified a novel lncRNA, WEE2-AS1, which was significantly upregulated at both the m^6^A modification and the transcription levels, and its high expression was correlated with a poorer prognosis in GBM. Functionally, we confirmed that WEE2-AS1 promoted GBM pathogenesis and malignant development in vitro and in vivo. Mechanistically, METTL3 mediated the m^6^A modification of WEE2-AS1 and enhanced its expression in an IGF2BP3-dependent manner. Moreover, WEE2-AS1 promoted RPN2 protein stabilization by preventing CUL2-mediated RPN2 K322 ubiquitination, thereby contributing to GBM malignant progression by activating the PI3K-Akt signaling pathway (Figure 6H). Furthermore, we found that blocking WEE2-AS1 expression improved the therapeutic sensitivity of dasatinib, a CNS penetrant that is FDA-approved for pediatric chronic myeloid leukemia, in GBM, providing a promising strategy for improving targeted combination therapy for GBM patients.

M^6^A is the most prevalent modification in eukaryotic lncRNAs, influencing nearly every stage of RNA metabolism, including splicing, decay, export and stabilization, whose function is mediated by specific m^6^A readers 38,39. In this study, we identified a new m^6^A-modified lncRNA, WEE2-AS1. In investigating the molecular mechanisms underlying the specific upregulation of WEE2-AS1 in GBM, we identified IGF2BP3, an m^6^A reader and a member of the IGF2BP family, which could promote the stability of target RNAs and thereby regulate their expression 40,41, playing a carcinogenic role in the malignant progression of GBM 22,42. The results from m^6^A-seq showed that the m^6^A site of WEE2-AS1 was in the exon 7 region, which was further verified by luciferase reporter assays (Figure 3). Further investigation showed that the IGF2BP3 protein recognizes the m^6^A site of WEE2-AS1 through its KH1-2 domain, thereby regulating its stability (Figure 3). These results suggested that the METTL3-mediated m^6^A modification of WEE2-AS1 promotes its expression by enhancing its stability in an IGF2BP3-dependent manner.

LncRNAs have a wide subcellular distribution in cells, and this characteristic determines the diversity of their functional mechanisms. Here, we demonstrated that WEE2-AS1, which is mainly distributed in the cytoplasm, promotes the growth, invasion and migration of GBM both in vitro and in vivo (Figure 2 and Figure S2-3), suggesting that WEE2-AS1 has a carcinogenic role in GBM. Further studies on its downstream functional mechanisms revealed that WEE2-AS1 can stabilize RPN2. As one of the major posttranslational protein modifications, ubiquitination mediates protein-specific degradation in eukaryotic cells and participates in and regulates almost all life activities, such as the cell cycle, signaling and DNA damage repair 26. E3 ubiquitin ligase is one of the most critical and heterogeneous enzymes in the ubiquitination pathway 27, and there are no reports of E3 ubiquitin ligase-mediated degradation of RPN2 ubiquitination. Our study showed that RPN2 can bind to CUL2, whose binding capacity can be attenuated by overexpression of WEE2-AS1. CUL2 is an essential component of the Cullin-RING ligase complex, which mediates binding between E2 enzymes (via RING proteins) and target proteins, which in turn degrade the target proteins 43,44. Zheng S, et al. 28 found that CUL2 protein levels are inversely related to those of HIF-1α, VEGF-A, Cyclin B1, and EGFR. Elevated CUL2 expression predicts increased radiosensitivity and dampened signal intensities in perfusion imaging in GBM, suggesting that CUL2 can be integrated as a potential biomarker in facilitating GBM prognosis and radiosensitivity profiling. In this study, we reveal another mechanism by which CUL2 promotes the malignant progression of GBM, re-emphasizing its significance as a protective prognostic factor for GBM patients.

The discovery of anticancer drugs has always been a significant area of cancer research. Although there is a substantial market for cancer treatment drugs and a large number of anticancer drugs in development, there are still relatively few anticancer drugs on the market, indicating that drug development is highly challenging. The best way to discover a new drug is to start with an old drug, which can greatly reduce the time, energy and financial resources of premarketing clinical trials and is a research direction with great potential 45,46. In this study, we found that WEE2-AS1 is negatively correlated with the bioavailability of dasatinib, a highly potent second-generation adenosine triphosphate-competitive inhibitor shown to be effective against multiple protein tyrosine kinases 30 that could cross the blood-brain barrier 32 and has been approved for pediatric chronic myeloid leukemia by the FDA 31. Several studies have demonstrated improved efficacy of dasatinib in the treatment of GBM, especially in GBM carrying the PDGFRA mutation 33-36. Recently, scRNA-seq data for dasatinib-resistant glioblastoma demonstrated increased AKT activation 37, which was also present in GBM cells with high WEE2-AS1 expression. Our further functional assays also confirmed that WEE2-AS1 enhances the efficacy of dasatinib in GBM in vitro and in vivo (Figure 6), providing a promising strategy for improving targeted combination therapy for GBM patients.

In summary, we identified WEE2-AS1 as a novel m^6^A-modified lncRNA that promotes GBM progression and is posttranscriptionally stabilized by IGF2BP3. Moreover, we confirmed that WEE2-AS1 promoted RPN2 protein stabilization by preventing CUL2-mediated RPN2 K322 ubiquitination, thereby contributing to GBM malignant progression by activating the AKT signaling pathway. Cumulatively, this work highlights that WEE2-AS1 may serve as a potential prognostic biomarker and therapeutic target in GBM, the knockdown of which significantly improved the efficacy of dasatinib, a CNS penetrant that is FDA-approved for pediatric chronic myeloid leukemia. Thus, WEE2-AS1 provids a promising strategy for improving targeted combination therapy for GBM patients.

## Methods and Materials

### Patients and specimens

Human GBM tissues and normal brain tissues (the cortex of decompressive surgery patients with brain trauma or hypertensive intracerebral hemorrhage) were obtained from patients admitted to Qilu Hospital from November 2017 to December 2019. All participants provided written informed consent, and the research was approved by the Ethical Committee on Scientific Research of Shandong University Qilu Hospital (approval number: KYLL-2018-324).

### Data acquisition

The Cancer Genome Atlas (TCGA) GBM RNA sequencing (RNA-seq) transcriptome data and corresponding clinicopathological parameters of GBM patients were obtained from the TCGA database (http://cancergenome.nih.gov/). The Wang RNA-seq dataset (FPKM format) and clinical information were extracted from the supplementary data of the article 20, and the missing data were obtained with the K-nearest neighbor (KNN) method. The m^6^A-seq data have been deposited in SRA PRJNA661159 (the data are being processed, submission ID: SUB8069560, released when the paper is published). The RNA-seq data of our local samples have been deposited in the Genome Sequence Archive (GSA) under accession number CRA002339. The processed data and basic association analyses will be made available in the supplementary data or from the corresponding author upon reasonable request.

### Cell culture

Human GBM cell lines (U251MG, U118MG, LN229 and A172) and the human embryonic kidney cell line 293T (HEK293T) were purchased from ATCC. Cells were maintained in DMEM supplemented with 10% fetal bovine serum. Normal human astrocytes (NHA) were obtained from Lonza (Walkersville, MD, USA) and cultured in Astrocyte Medium (ScienCell; Carlsbad, CA, USA) supplemented with the Astrocyte Growth Medium BulletKit (ScienCell). All patient-derived GSC cell lines, including mesenchymal (MES)-subtype GSC cell lines (GSC20 and GSC267), proneural (PN)-subtype GSC cell lines (GSC11 and GSC8-11), and neural progenitor cells (NPCs), were kindly donated by Dr. Frederick F. Lang and Dr. Krishna P.L. Bhat (The University of Texas, M.D. Anderson Cancer Center, Houston, TX, USA). The cells were cultured in DMEM/F12 supplemented with B27 (Invitrogen, California, USA), 20 ng/ml EGF (R&D Systems, USA), and 20 ng/ml bFGF (R&D Systems, California, USA). Cells were cultured in a standard humidified atmosphere of 5% CO2 at 37 °C.

### Western blot analysis

Protein was extracted from GSC cells or GBM cells. Primary antibodies to the following were used: GAPDH (Cell Signaling Technology, 5174), β-actin (Cell Signaling Technology, 14074), N-cadherin (Cell Signaling Technology, 13116), E-cadherin (Proteintech, 20874-1-AP), CD44 (Proteintech, 15675-1-AP), AKT (Cell Signaling Technology, 9272), phospho-Akt (Ser473, Cell Signaling Technology, 4060), CDK1/cdc2 (Cell Signaling Technology, 9116), phospho-CDK1/cdc2 (Tyr15) (Cell Signaling Technology, 4539), cyclin B1 (Cell Signaling Technology, 12231), P21 (Cell Signaling Technology, 2947), IGF2BP3 (Abcam, ab177477), ubiquitin (Cell Signaling Technology, 3936), DYKDDDDK Tag (Cell Signaling Technology, 14793), His-tag (Cell Signaling Technology, 12698), CUL2 (Proteintech, 10981-2-AP; Santa, sc-166506), and RPN2 (Abcam, ab244399).

### RNA interference and lentivirus transfection

Transient knockdown of WEE2-AS1, IGF2BP3, RPN2 and CUL2 was achieved using small interfering RNAs (siRNAs) from GenePharma (Shanghai, China) or RiboBio (Guangzhou, China) following the protocol of Lipo3000 (L300015, Invitrogen, USA).

Human full length WEE2-AS1 sequence and control sequence were cloned into the GV502 lentiviral vector to construct lentiviruses for stable overexpression (Genechem, China). While, the sequences of WEE2-AS1 knockdown (shRNA), and the corresponding scramble control (shNC) were cloned into GV112 lentiviral vector to construct lentiviruses (Genechem, China). The detailed oligonucleotide sequence used in this study are shown in Table S1-1.

### RNA extraction and RT-PCR

TRIzol (Invitrogen, Carlsbad, CA, USA) was used to extract total cellular RNA according to the manufacturer's protocol. The primers used are shown in Table S1-3. Quantitative PCR was performed using TB green Premix Ex Taq (Takara; Tokyo, Japan) on a Real-Time PCR Detection System (480II, Roche; Basel, Switzerland).

### Luciferase reporter assay

Luciferase assays were performed using reporter lysis (Catalog #E3971, Promega, USA) and luciferase assay reagents according to the manufacturer's instructions. In brief, pmiRGLO-WT-WEE2-AS1, pmiRGLO-MUT1-WEE2-AS1 and pmiRGLO-MUT2-WEE2-AS1 (Bioscience., Jinan, China) were transfected with U251MG or GSC267 cells. After 24 h of incubation, the cells were analyzed with the Dual-Glo Luciferase Assay System (Promega) according to the manufacturer's instructions. Detailed source information for vectors is available in Table S1-2.

### Biotin-labeled RNA pulldown assay and mass spectrometry analysis

WEE2-AS1 cDNAs (sense and antisense; Bioscience Technology, Jinan, China) were transcribed in vitro according to the manufacturer's instructions for the TranscriptAid T7 High Yield Transcription Kit (Thermo Fisher Scientific K0441). Then, the transcripts were labeled with biotin using the RNA 3' End Desthiobiotinylation Kit (Thermo Fisher Scientific) to generate RNA probes. Finally, an RNA pulldown assay was performed according to the manufacturer's instructions for the Magnetic RNA-Protein Pull-Down Kit (Thermo Fisher Scientific). Eluted proteins were detected by Western blotting and mass spectrometry analysis. Detailed source information for vectors is available in Table S1-2.

### Co-IP and mass spectrometry analysis

Co-IP was performed as previously described 47 with ubiquitin (Cell Signaling Technology, 3936), DYKDDDDK Tag (Cell Signaling Technology, 14793), His-Tag (Cell Signaling Technology, 12698), and RPN2 (Abcam, 244399) antibodies. Bead-bound proteins were released and analyzed by Western blotting and mass spectrometry analysis. The full-length and mutant plasmids of RPN2 were synthesized and subcloned into the pcDNA3.1-3XFlag vector. Detailed source information for vectors is available in Table S1-2.

### RNA-binding protein immunoprecipitation (RIP) assay

RIP assays were performed according to the EZ-Magna RIP RNA-binding Protein Immunoprecipitation Kit (Merck Millipore) with m6A (Synaptic Systems, #202003/202011), IGF2BP3 (Abcam, ab177477) and RPN2 (Proteintech, 10576-A-AP) antibodies. Coprecipitated RNAs were eluted and purified and then detected by qRT-PCR. Total RNA (input) served as the internal control. For the IGF2BP3 RIP assay, human full-length IGF2BP3 cDNA and truncated cDNA were subcloned into the pcDNA3.1 vector (Bioscience, Jinan, China). Detailed source information for vectors is available in Table S1-2.

### Animal studies

Luciferase-expressing human GBM cell lines were randomly injected into the frontal lobes of 4-week-old BALB/c nude mice (5×10^5^ cells in 10 μl of PBS) using a stereotactic apparatus to build a xenograft model. Tumor growth was examined at days 7, 14 and 21 using bioluminescence imaging (IVIS spectrum in vivo imaging system, PerkinElmer; Hopkinton, MA, USA). Mouse brains were harvested through H&E and IHC staining. For combined drug animal experiments, dasatinib (HY-10181, MCE) was dissolved in 90% SBE-β-CD (C871854, MACKLIN) in saline. Mice were gavaged daily at a concentration of 10 mg/kg. All procedures that involved mice were approved by the committee and laboratory animal department of the Qilu Hospital of Shandong University (Institutional Animal Care and Use Committee issue No. DWLL-2021-039).

### Statistical analysis

Statistical analyses were performed using GraphPad Software 8 (GraphPad Software Inc., CA, USA). Correlations between variables were explored using Pearson or Spearman coefficients. The Kaplan-Meier method was used to generate survival curves for the subgroups in each dataset, and the log-rank (Mantel-Cox) test was used to determine if they were significantly different. The optimal cutoff point for survival information in the Wang dataset was evaluated based on the association between survival time and the expression of WEE2-AS1 using the “survminer” package. The hazard ratios (HRs) for the univariate analyses were calculated using a univariate Cox proportional hazards regression model. The univariate analysis results for prognosis were visualized using the “forestplot” R package. All data are expressed as the means ± standard deviations (SDs). Student's t test was used for two-group comparisons. For comparisons among more than two groups, the Wilcoxon test and one-way ANOVA were used for nonparametric and parametric data. P > 0.05 was considered to indicate nonsignificance (ns), and P < 0.05 was considered to indicate statistical significance (*P < 0.05; **P < 0.01; ***P < 0.001, ****P < 0.0001). All data processing with R packages was performed using R Studio (version 3.6.3).

## Supplementary Material

Supplementary materials and methods, figures and tables.

## Abbreviations

GBM: Glioblastoma
FISH: Fluorescence in situ hybridization
H&E: Hematoxylin and eosin
IHC: Immunohistochemistry
RBP: RNA binding protein
m6A: N6-methyladenosine
m6A-seq: m6A high-throughput sequencing
lncRNAs: Long non-coding RNAs
RIP: RNA immunoprecipitation
CNS: Central Nervous System
qRT-PCR: Quantitative real-time polymerase chain reaction
TCGA: The Cancer Genome Atlas
IGF2BP3: Insulin like growth factor 2 mRNA binding protein 3
RPN2: ribophorin II
