# Supplementary material for: The N6-methyladenosine-mediated lncRNA WEE2-AS1 promotes glioblastoma progression by stabilizing RPN2
Source: Theranostics. 2022 Aug 29;12(14):6363–79. doi: 10.7150/thno.74600 (PMC9475453; doi:10.7150/thno.74600)
Supplement: Supplementary file 1 — Supplementary materials and methods, figures and tables. [file thnov12p6363s1.zip › Supplementary Figure and method and Table S1.docx]

**Supplementary Figure**

**The N^6^-methyladenosine-mediated lncRNA WEE2-AS1 promotes glioblastoma progression by stabilizing RPN2**

# Authors and affiliations:

*Boyan Li1,2#, Rongrong Zhao1,2#, Wei Qiu1,2, Ziwen Pan1,2, Shulin Zhao1,2, Yanhua Qi1,2, Jiawei Qiu1,2, Shouji Zhang1,2, Qindong Guo1,2, Yang Fan1,2, Hao Xu1,2, Ming Li1,2, Gang Li1,2*, Hao Xue1,2**

1. Department of Neurosurgery, Qilu Hospital, Cheeloo College of Medicine and Institute of Brain and Brain-Inspired Science, Shandong University, Jinan 250012, Shandong, China;

2. Shandong Key Laboratory of Brain Function Remodeling, Jinan 250012, Shandong, China.

***Boyan Li***^1,2#^, ***Rongrong Zhao***^1,2#^ contributed equally to this work.

* Correspondence:

***Gang Li***, Department of Neurosurgery, Qilu Hospital of Shandong University, 107 Wenhua Western Road; Jinan, Shandong 250012, China, E-mail: dr.ligang@sdu.edu.cn; Tel: 086-0531-82166615.

***Hao Xue***, Department of Neurosurgery, Qilu Hospital of Shandong University, 107 Wenhua Western Road; Jinan, Shandong 250012, China, E-mail: xuehao@sdu.edu.cn; Tel: 086-0531-82166615


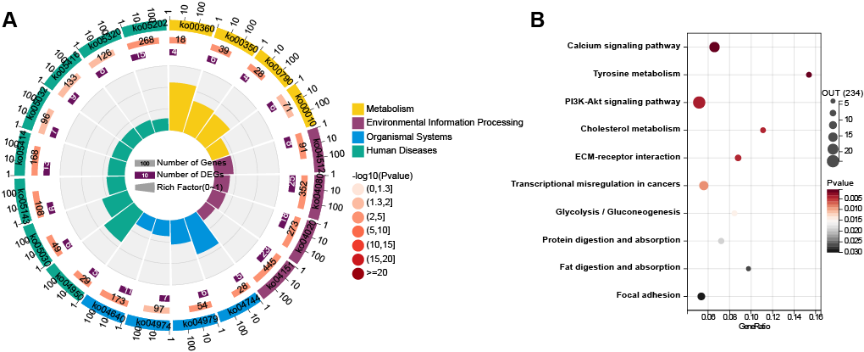


**Supplementary Figure1 Potential biological role of WEE2-AS1 in the progression of GBM. (A)** Summary of KEGG enrichment class, and (**B**) function terms on upregulated genes in WEE2-AS1 high, compared with low group samples in TCGA GBM dataset.


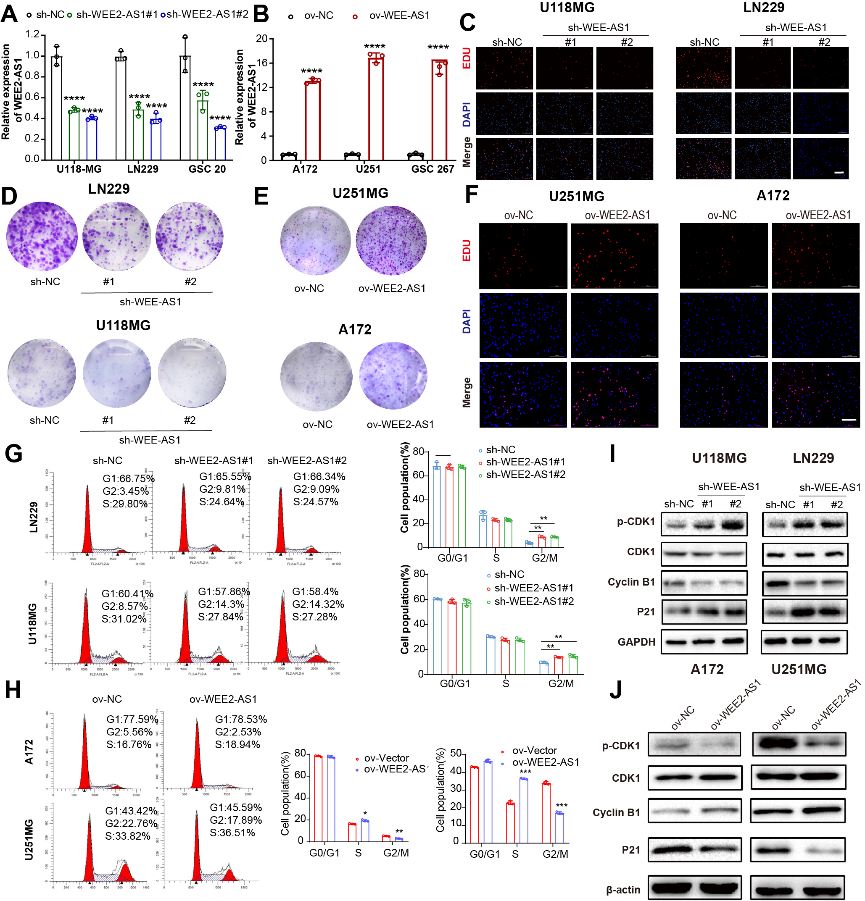


**Supplementary Figure2** **WEE2-AS1 promotes proliferation, migration and invasion of GBM cells in vitro.** qRT-PCR assays showing the relative expression in GBM cells and GSCs transfected with (**A**) knocking down WEE2-AS1 (sh-WEE2-AS1) or corresponding negative contorl (sh-NC), and (**B**) overexpressing WEE2-AS1 (ov-WEE2-AS1) or corresponding negative control (ov-NC). **C** EDU assays showing the proliferation ability of GBM cells transfected with sh-NC or sh-WEE2-AS1, scale bar, 50μm. Colony-forming assays showing the proliferation ability of GBM cells transfected with (**D**) sh-NC or sh-WEE2-AS1, and (**E**) ov-NC or ov-WEE2-AS1. **F** EDU assays showing the proliferation ability of GBM cells transfected with ov-NC or ov-WEE2-AS1, scale bar, 50μm. Flow Cytometry assays showing the cell cycle progression of GBM cells transfected with (**G**) sh-WEE2-AS1 or sh-NC, and (**H**) ov-WEE2-AS1 or ov-NC. Quantification histogram represented cell population. Data represented mean ± SD from at least three independent experiments. Western blot assays showing the protein expression of p-CDK, CDK, CyclinB1 and p21 expression in GBM cells transfected with (**I**) sh-NC or sh-WEE2-AS1, and (**J**) ov-NC or ov-WEE2-AS1. Data represented mean ± SD from at least three independent experiments. The statistical significance is shown as: *P < 0.05; **P < 0.01; ***P < 0.001; ****P < 0.0001.


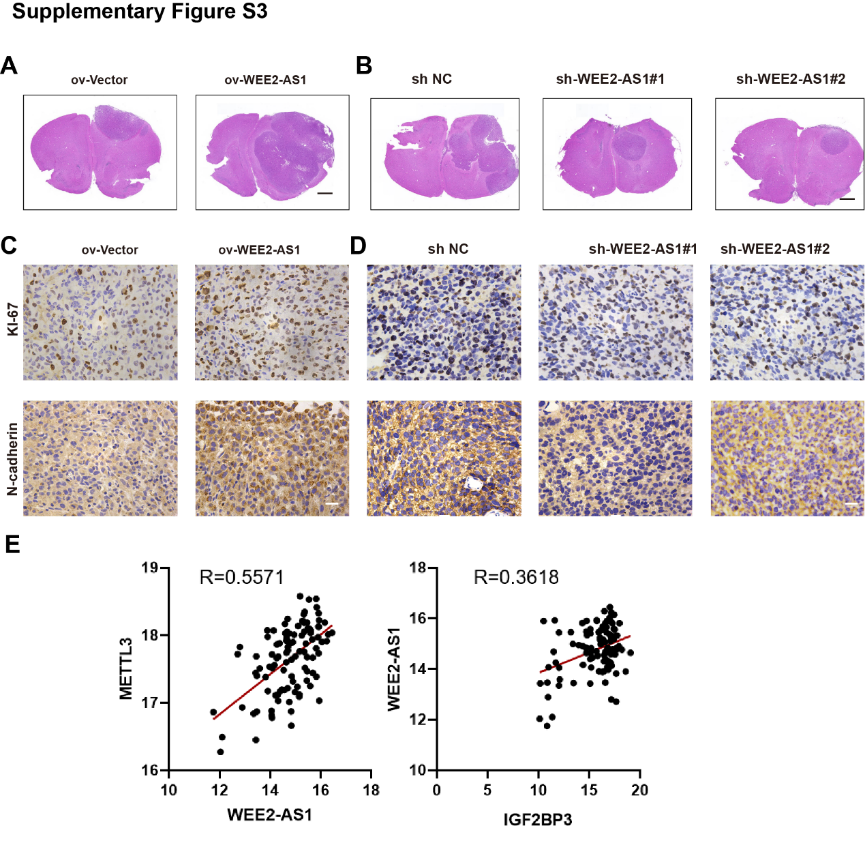


**Supplementary Figure3 WEE2-AS1 promotes proliferation, migration and invasion of GBM cells in vivo.** Representative HE staining images of sections from xenograft mice implanted with luciferase-labeled (**A**) U251MG expressing ov-WEE2-AS1 or ov-NC, and (**B**) LN229 expressing sh-WEE2-AS1 or sh-NC in the indicated time (n≥4), scale bar, 1000μm. Representative KI-67 and N-cadherin immunohistochemistry (IHC) staining images of sections from xenograft mice implanted with luciferase-labeled (**C**) U251MG expressing ov-WEE2-AS1 or ov-NC, and (**D**) LN229 expressing sh-WEE2-AS1 or sh-NC in the indicated time (n≥4), scale bar, 50μm.

**E** The correlation between the METTL3/IGF2BP3 and WEE2-AS1 in glioma tissues from the Wang database.


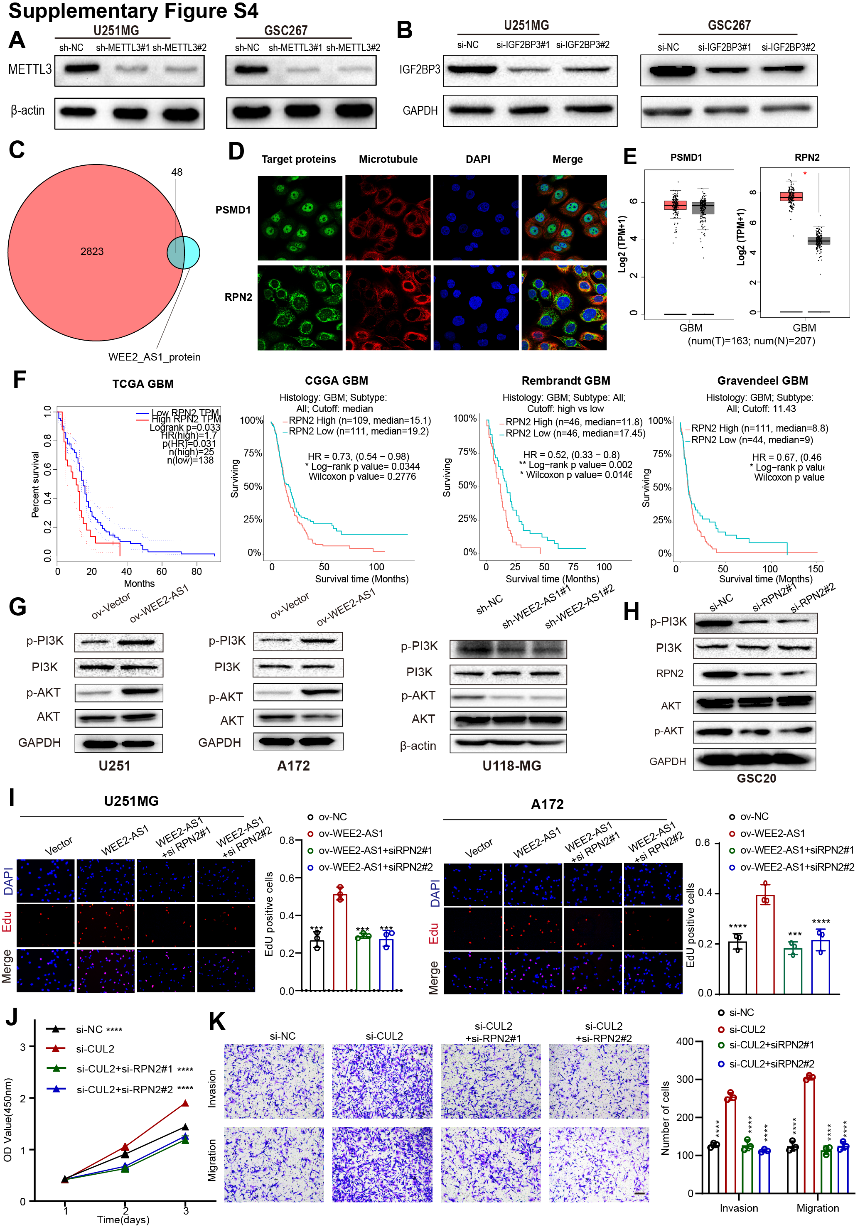


**Supplementary Figure4 WEE2-AS1 promotes RPN2 protein stabilization to promote GBM malignant progression.** Western blot assays showing the protein expression of (**A**) METTL3 in GBM cells transfected with si-NC or si-METTL3, and (**B**) IGF2BP3 in GBM cells transfected with si-NC or si-IGF2BP3. **C** Venn diagram showing the overlapping of interacted proteins with circNEIL3 between our mass spectrometric data and RBPs. **D** Immunofluorescence assays showing the localization of PSMD1 and RPN2 proteins downloaded from The Human Protein Atlas database (https://www.proteinatlas.org/). **E** GEPIA database showing the relative expression of PSMD1 and RPN2 in TCGA GBM samples, compared with GETx NBTs. **F** Kaplan–Meier survival curves showing that RPN2 is a prognostic risk factor in TCGA, CGGA, Rembrandt and Gravendeel GBM datasets. Log-rank test analysis was used. Western blot assays showing the AKT and PI3K phosphorylation level in GBM cells transfected with (**G**) ov-NC or ov-WEE2-AS1, sh-NC or sh-WEE2-AS1, and (**H**) si-NC or si-RPN2. **I** EDU assays showing the proliferation ability of GBM cells co-transfected with ov-NC or ov-WEE2-AS1, and siRPN2 as indicated, scale bar, 50μm. Quantification histogram represented EDU positive cells. Data represented mean ± SD from at least three independent experiments. **J** CCK8 assays showing the proliferation ability of GBM cells co-transfected with si-NC or si-CUL2, and siRPN2 as indicated. **K** Representative transwell migration and invasion assays showing the migration and invasion ability of GBM cells co-transfected with si-NC or si-CUL2, and siRPN2 as indicated, scale bar, 200um. Quantification histogram represented cell numbers. Data represent mean ± SD from at least three independent experiments. The statistical significance is shown as: *P < 0.05; **P < 0.01; ***P < 0.001; ****P < 0.0001.


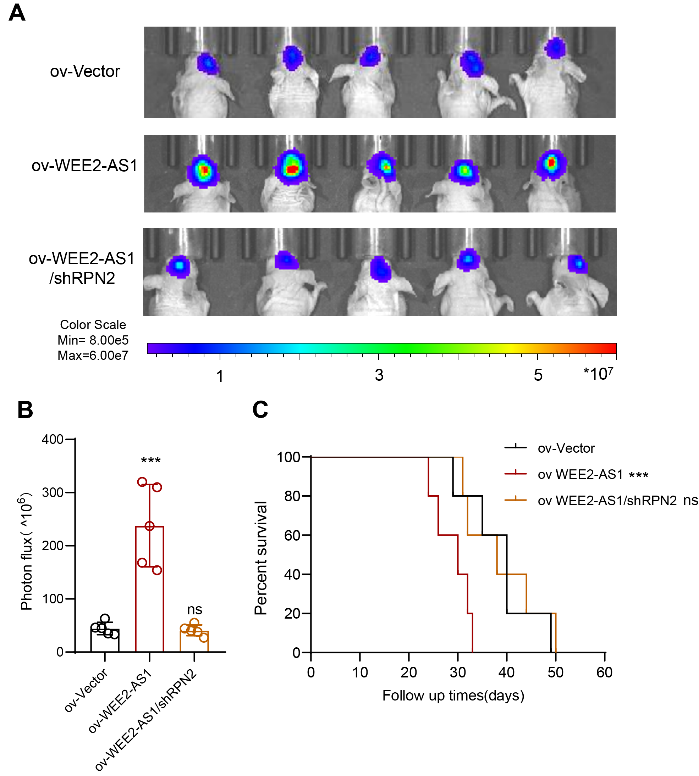


**Supplementary Figure 5 RPN2 rescue the malignant progression of WEE2-AS1 in vivo**

**A** Bioluminescent image showing the tumor size of mice implanted with luciferase-labeled LN229 cells expressing ov-vector or ov-WEE2-AS1 and ov-WEE2-AS1/shRPN2 for the indicated times. **B** The quantification histogram represents the bioluminescent flux. Data represent the mean ± SD, n=5 for each group. **C** Kaplan–Meier survival curves for mice implanted with luciferase-labeled LN229 cells expressing ov-vector or ov-WEE2-AS1 and ov-WEE2-AS1/shRPN2. Log-rank analysis was used, n=5 for each group.

**Supplementary Methods**

# m^6^A-seq and data analysis

m6A antibody-enriched RNA was sequenced by Novogene (Beijing, China). In brief, a total of 300 µg of RNA was extracted from GBM or normal brain tissues. The integrity and concentration of the extracted RNAs were detected using an Agilent 2100 bioanalyzer (Agilent) and a simpliNano spectrophotometer (GE Healthcare), respectively. Fragmented enriched RNA (~100 nt) was incubated for 2 h at 4°C with anti-m6A polyclonal antibody (Synaptic Systems) in the immunoprecipitation experiment. Then, immunoprecipitated RNAs or input were used for library construction with a NEBNext ultra RNA library preparation kit for Illumina (New England Biolabs). The library preparations were sequenced on an Illumina NovaSeq or HiSeq platform with a paired-end read length of 150 bp according to the vendor’s recommended protocol. Sequencing was carried out with 3 independent biological replicates for each group.

# Differential expression analysis and functional analysis

Differential analysis of lncRNA expression between GBM and normal brain tissues was performed using the “DESeq2” R package. For differential analysis of mRNA expression between the WEE2-AS1 high and low groups, we first classified patients into two groups based on the median cutoff of WEE2-AS1 expression in the TCGA GBM dataset. Then, differential expression analysis was analyzed using the “limma” R package. Furthermore, upregulated genes were analyzed in the Kyoto Encyclopedia of Genes and Genomes (KEGG) database[23].

To explore the biological behaviors among these distinct RPN2 expression samples, we used some gene sets of HALLMARK^[24]^ from the MSigDB database to estimate pathway enrichment scores for each sample for GSVA enrichment analysis using the “GSVA” R package. Gene set enrichment analysis (GSEA) was also used to estimate the enrichment of various biological processes in each sample.

# Cell counting kit-8 (CCK-8) assay

The CCK-8 (CK04, Dojindo; Rockville, MD) assay was carried out according to the manufacturer’s protocol. In brief, cells were incubated in 96-well plates for different times. CCK-8 solution (10 μL) was added to each well, and the plates were incubated for 1 h at 37°C and finally measured in a microplate reader.

# Neurosphere formation assay

GSC tumor spheres were digested into single cells by Accutase solution (A6964, Sigma-Aldrich, USA) and implanted into 6-well plates at 1000 cells per well, cultured for 1-2 weeks and imaged with a Leica microscope.

# Extreme limiting dilution assay (ELDA)

GSCs were implanted into 96-well plates according to a density gradient of 0, 2, 4, 8, 16, 32 and 64 cells per well in 10 replicates. The number of wells that successfully formed tumor spheres was counted on the seventh day.

# Cell migration and invasion assays

Transwell assays were performed as previously described[20]. A 3D tumor spheroid invasion assay was performed using a 96-well 3D spheroid BME cell invasion assay kit (3500-096-K, Trevigen, USA) according to the manufacturer’s protocol. In brief, GBM cells were spread in ECM medium for 72 h to promote tumor spheroidization, and invasion matrix was added to the medium and photographed at different time points.

# RNA-FISH

RNA-FISH was performed according to the manufacturer’s protocol (RioboBio, Guangzhou, China). In brief, cells were incubated with 20 nM of a Cy3-labeled WEE2-AS1 FISH probe in hybridization buffer at 73°C for 12 h and then incubated at 37°C for 12 h. Finally, the cells were stained with DAPI and imaged under confocal microscopy (Leica SP8).

# Protein stability assay

Cycloheximide (25 µg/mL; MCE, HY-12320) was added to the culture medium to inhibit translation, and cell lysates were prepared at different hours to observe the protein by Western blotting.

# RNA stability assay

Actinomycin D (5 μg/ml, ActD, MCE, HY-17559) was used to test the RNA stability of GBM cells. The half-life of RNA was analyzed by collecting RNA from cells at different time points, and GAPDH was used for normalization.

**Supplementary Table S1-1. Oligonucleotide sets used in this study**

| **siRNAs or shRNAs** | **Sequences** |
| --- | --- |
| si-NC | 5'-UUCUCCGAACGUGUCACGUTT-3' |
| si-*WEE2-AS1#*1 | 5'- GCAGACCAUUGUAAUUCAUTT-3' |
| si-*WEE2-AS1#2* | 5'- GCGAUGAAUCCAAUGAUAATT-3' |
| si-*RPN2*#1 | 5'- GAGTCAAGATCTCCACTGA -3' |
| si-*RPN2#2* | 5′- CGACTGTCTTGTCCCAGAA -3′ |
| si-*RPN2#3* | 5′- CCTACAAGCTCATGGATCA -3′ |
| si-IGF2BP3#1 | 5'-CCUUGAAAGUAGCCUAUAUTT-3' |
| si-IGF2BP3#2 | 5'-GCAGGAAUUGACGCUGUAUTT -3' |
| sh-METTL3#1 | 5′- GCACTTGGATCTACGGAAT-3′ |
| sh-METTL3#2 | 5′- GCTGCACTTCAGACGAATTAT-3′ |
| si-CUL2#1 | 5′-GGATAAGGCCCTTACGTCA-3′ |
| si-CUL2#1 | 5′- GCCCTTACGTCAGTTGTAA-3′ |
| sh-*WEE2-AS1#1* | Designed and constructed by GeneChem (Shanghai, China) Technology |
| sh-*WEE2-AS1#2* | Designed and constructed by GeneChem (Shanghai, China) Technology |
| sh-NC | 5'-TTCTCCGAAGGTGTCACGG-3' |

**Supplementary Table S1-2. Plasmids used in this study**

| **Plasmids** | **Resources** |
| --- | --- |
| pcDNA3.1-T7 promoter-*WEE2-AS1* and its antisense | Bioscience (Jinan,China) |
| pcDNA3.1-3xFlag-empty vector | Bioscience (Jinan,China) |
| pcDNA3.1-3xFlag-IGF2BP3- full length | Bioscience (Jinan,China) |
| pcDNA3.1-3xFlag-IGF2BP3- delet KH1/2 | Bioscience (Jinan,China) |
| pcDNA3.1-3xFlag-IGF2BP3- delet KH3/4 | Bioscience (Jinan,China) |
| pcDNA3.1-3xFlag-IGF2BP3- RRM1/2 | Bioscience (Jinan,China) |
| pcDNA3.1-3xFlag-IGF2BP3- KH1/2 | Bioscience (Jinan,China) |
| pcDNA3.1-3xFlag-IGF2BP3- KH3/4 | Bioscience (Jinan,China) |
| pcDNA3.1-3xFlag-RPN2-full length | WZbio (Jinan,China) |
| pcDNA3.1-3xFlag-RPN2-Mut K69R | Bioscience (Jinan,China) |
| pcDNA3.1-3xFlag-RPN2-Mut K70R | Bioscience (Jinan,China) |
| pcDNA3.1-3xFlag-RPN2-Mut K154R | Bioscience (Jinan,China) |
| pcDNA3.1-3xFlag-RPN2-Mut K306R | Bioscience (Jinan,China) |
| pcDNA3.1-3xFlag-RPN2-Mut K311R | Bioscience (Jinan,China) |
| pcDNA3.1-3xFlag-RPN2-Mut K322R | Bioscience (Jinan,China) |
| pcDNA3.1-3xFlag-RPN2-Mut K627R | Bioscience (Jinan,China) |
| pcDNA3.1-His-Ubiquitin -full length | Bioscience (Jinan,China) |
| pmiRGLO-WT-WEE2-AS1 | Bioscience (Jinan,China) |
| pmiRGLO-Mut1-WEE2-AS1 | Bioscience (Jinan,China) |
| pmiRGLO-Mut2-WEE2-AS1 | Bioscience (Jinan,China) |

**Supplementary Table S1-3. Primer sets used in this study**

| **Primer set** | **Primers** | **Sequence (5’-3’)** |
| --- | --- | --- |
| *WEE2-AS1* | F  R | 5'-CACAGGGGGAAAGCGAAGTT-3'  5'-TGTGTCTGCAAGGGGAGAAC -3' |
| *GAPDH* | F  R | 5'-GCACCGTCAAGGCTGAGAAC-3'  5'-TGGTGAAGACGCCAGTGGA-3' |
| *β-actin* | F  R | 5'-CATGTACGTTGCTATCCAGGC -3'  5'-CTCCTTAATGTCACGCACGAT-3 |
| *METTL3* | F  R | 5'-TAAGCCCAGCACAGCTTCAG -3'  5'-CGTGGAGATGGCAAGACAGA -3' |
| *RPN2* | F  R | 5'-TGCCAATGTCTCCAACTTCACT-3'  5'-CCCAGCATAGCAGCATGTCC-3' |
| IGF2BP3 | F  R | 5'-GCACTTCCCTTTGTTGTAGTC-3'  5'-AGCACTTCCCTTAGGTTACTC-3' |
